# Positive *Francisella tularensis* meningitis outcome despite delayed identification: a case report

**DOI:** 10.1186/s12941-023-00642-7

**Published:** 2023-10-24

**Authors:** Vesa Mäki-Koivisto, Marianne Korkala, Lotta Simola, Sonja Suutari-Kontio, Sini Koivunen, Teija Puhto, Ilkka S. Junttila

**Affiliations:** 1 NordLab, Oulu, Finland; 2 Oulu University Hospital, Oulu, Finland; 3 University of Oulu, Oulu, Finland; 4 Tampere University, Tampere, Finland; 5 Fimlab Laboratories, Tampere, Finland

**Keywords:** *Francisella tularensis*, Meningitis, Maldi-tof, PCR, Ciprofloxacin

## Abstract

*Francisella tularensis* is a Gram-negative bacteria, that may cause a zoonotic disease, tularemia. Here, we describe a patient case, where a previously healthy young woman in Northern Finland contacted health care because of fever and headache. Due to the symptoms and lack of further diagnostic tools in primary health care, she was transferred to University Hospital (UH) where ampicillin and ceftriaxone was given empirically. A cerebrospinal fluid sample (CSF) was drawn showing small Gram-negative rods that grew on chocolate agar after 2 days of incubation. Matrix-assisted laser-desorption-ionization time of-flight (Maldi-tof) did not provide identification, but the bacteria was interpreted as sensitive to ciprofloxacin and the treatment was changed to ciprofloxacin. During the time the patient was infected, there were several positive tularemia samples found in the area. Therefore, an in house tularemia nucleic acid method (PCR) was used on the bacterial culture. Additionally, 16S rDNA sequencing was performed and these methods identified the bacteria as *F. tularensis*. Fortunately, the patient recovered completely with ciprofloxacin and was discharged without any complications. Our case underlines the need to understand the limits of specific diagnostic methods, such as Maldi-tof, used in clinical laboratory settings. It also highlights the need of both clinicians and laboratory staff to be aware of the many clinical presentations of tularemia when working in an endemic area.

## Background

*Francisella tularensis* is an aerobic Gram-negative intracellular coccobacilli and the causative agent of a rare zoonotic disease, tularemia, in humans [3]. Only a few cases of tularemia meningitis have been reported [7, 9, 12, 16, 24], but patient outcome may be fatal. According to Hofinger et al. [12] 7/16 cases were lethal during 1931 to 2009 in the USA, where *F. tularensis *spp*. tularensis* is more prevalent [2].

**Fig. 1:**
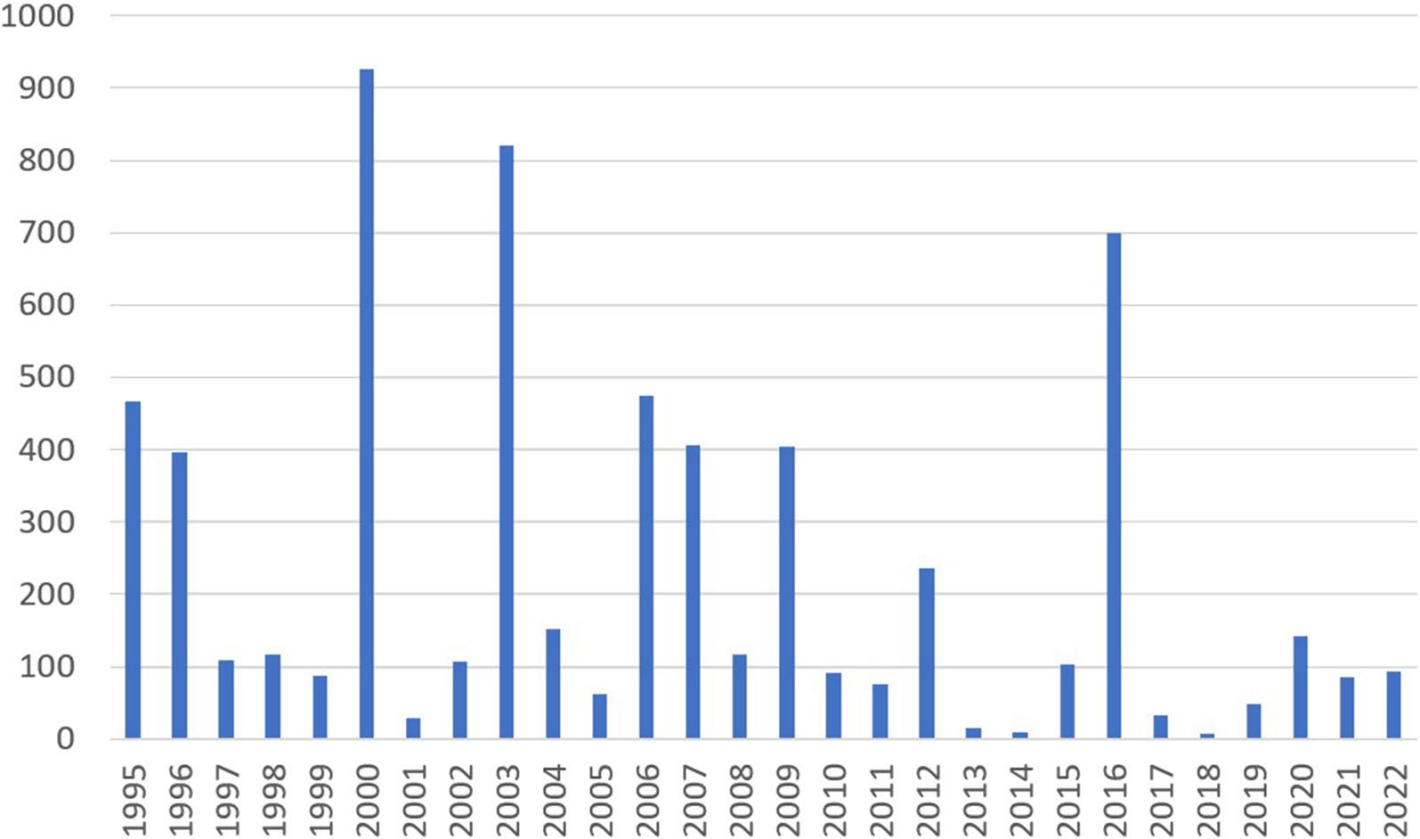
Annual cases of Tularemia in Finland during 1995–2022

*Francisella tularensis* causes infections on animals, mainly in hares, squirrels, mice, and other rodents [1]. The prevalence varies among animal populations, and the bacterium may remain contagious in soil, water and animal carcasses for months [23]. In the USA most cases are reported in the Pacific Northwest and South-Central parts. (https://www.cdc.gov/tularemia/index.html). In Europe, roughly 800 cases are reported annually with Sweden and Finland being the two countries with highest reported number of cases. Outside laboratory settings (occupational exposure, [16], four main routes of transmission for *F. tularensis* has been described; contaminated food/water, handling of infected animals, insect bites and aerosol (https://www.ecdc.europa.eu/en/tularaemia). In Sweden and Finland transmission to humans occurs most commonly via insect (haematophagosus arthropods) bites and accordingly, the incidence of tularemia in Finland is highest between July and October. No transmission between humans for example via aerosol or arthropods has been described [21, 23]. The prevalence of Tularemia in Finland has showed annual variation (Fig. 1), with annual cases exceeding the average prevalence in the years 1996, 2000, 2003, 2007, 2009 and 2016 (Finnish Institute for health and welfare). According to Finnish national legislation (Communicable Diseases Act and Decree) all clinical *F. tularensis* findings are ae reported to Finnish National Infectious Diseases Register maintained by Finnish Institute for Health and Welfare which keeps track on national epidemiological situation. However, no national reference laboratory that would confirm the subtype, or verify results of susceptibility testing exists https://sampo.thl.fi/pivot/prod/fi/ttr/shp/fact_shp?row=area-12260&column=time-12059&filter=reportgroup-12231).

*Francisella tularensis* poses a diagnostic challenge as it may be difficult to isolate from clinical samples [18]. Detection by culture-based methods is fastidious and requires special knowledge (and selective media). Serologic tests detecting *F. tularensis* specific antibodies are available, but may not be useful in an acute setting as the immune response may take days or even weeks to develop. Nucleic acid-based tests may be used in diagnostics, but commercial applications remain sparse and with various limitations [18]. Overall, these diagnostic challenges may contribute to an underestimation of the number of tularemia cases.

## Case presentation

A 28 year old female previously healthy with active outdoor life style got ill with fever, headache and neck pain. She had not been traveling recently or consumed abnormal diet. Three days later she sought her occupational health care unit where her CRP was found normal. It was assumed she had a minor viral infection and was discharged. After an additional two days her headache worsened with fever remaining high. She felt disoriented and vomited a few times. She also experienced vision impairment and contacted a primary health care emergency ward in a small town. A nasopharyngeal sample tested negative for Influenza A, Influenza B, Respiratory Syncytial Virus and Sars-CoV-2 with PCR method (GeneXpert, Cepheid). At this stage, the CRP was 49 mg/l with no leukocytosis. Upon neurological examination her vision was reduced and she had neck pain without stiffness.

**Fig. 2:**
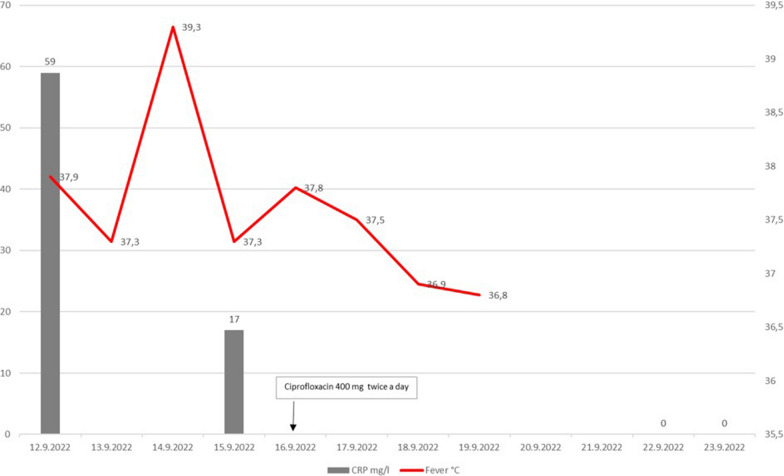
CRP and body temperature of patient during infection

She was transferred to Oulu UH where she was nauseous and suffered slight slowness in motoral movement. Upon examination her right pupil was slightly enlarged and the visual field seemed to be restricted in all directions. She did not have adenopathy, splenomegaly, any skin lesions or ulcers. Cranial computed tomography findings were normal. CRP was slightly elevated to 59 mg/l (Fig. 2). A CSF sample showed elevated leukocytes (218 × 10^6^/l) with 75% mononuclear cells, normal glucose (3.3. mmol/l (2.2–4.2 mmol/l)) and slightly elevated protein (525 mg/l (< 450 mg/l)). Therefore, encephalitis was suspected, and intravenous acyclovir was initiated. In addition, a CSF Gram stain showed small Gram-negative small rods under light microscopy, for this, dexamethasone ceftriaxone and ampicillin were added to empiric treatment.

The CSF was analyzed with a meningitis/encephalitis PCR method (BioFire® Filmarray® Meningitidis/Encephalitis panel) and found negative for all included microbes. Head MRI indicated no clear pathological findings. Electroencephalogram (EEG) demonstrated mild generalized slowing, but no signs of epileptic activity. The fever continued and her headache fluctuated.

Simultaneously with CSF, blood cultures were drawn upon admission to Oulu UH, and subsequently every 12 h. All blood cultures drawn were performed using Bact Alert system by BioMerieux. All blood cultures were incubated for 5.5 days [11] and they all remained negative.

The visual impairment, fever and headache continued for the patient. After two days of incubation, CSF cultures on chocolate agar (with vitox P05090A, Thermo Fischer) showed small grey/white colonies which were weakly catalase positive and oxidase negative, but Matrix-assisted laser-desorption-ionization time of-flight (Maldi-tof, VitekMS, BioMérieux) gave no identification. Antibiotic susceptibility of the colonies were performed initially using disk diffusion method. The strain was resistant (= 06 mm) to amoxicillin clavulanic acid, aztreonam, ertapenem, ceftazidim, ceftriaxone, cefuroxime and piperacillin/tazobactam. However, ciprofloxacin, tetracycline (both = 30 mm) and tobramycin (= 22 mm) showed clear inhibition of bacterial growth. For this, MIC E-test for ciprofloxacin was performed (MIC = 0.016 mg/L). Ampicillin was changed to ciprofloxacin (400 mg twice a day), but ceftriaxone and acyclovir were continued at this stage. Upon ciprofloxacin treatment, patient fever and headache disappeared and CRP was normalized and she felt normal.

Simultaneously, an in-house PCR based on the work by Skottman et al. [22] was performed on the bacterial culture, due to a high number of *F. tularensis* antibody positive persons the same week. The result was positive for *F. tularensis*. 16SrDNA sequencing of bacterial culture later confirmed the finding. According to CLSI guideline, E-test result for ciprofloxacin indicated the strain was sensitive to ciprofloxacin. Our laboratory routinely follows EUCAST recommendations for antimicrobial sensitivity testing, but as EUCAST has not provided interpretation for *F. tularensis* CLSI guidelines were used [6]. Based on the PCR result, acyclovir and ceftriaxone were discontinued and ciprofloxacin dosage was raised from 400 mg twice a day to 600 mg twice a day to make sure that level in cerebrospinal fluid was sufficient. The patient was discharged from UH after 8 days to her local regional hospital for intravenous treatment. Ciprofloxacin treatment was given for 14 days altogether and she recovered completely.

## Conclusions

Meningitis caused by *F. tularensis* is rare, but cases have been reported [7, 9, 12, 16, 24]. In our case *F. tularensis* was identified as the causative microbe by an in house PCR method and 16s rDNA sequencing. An assumption of subspecies *holarctica* as a causative agent was made based on the known geographical distribution of *F. tularensis* subspecies in Europe and Finland and the lack of recent traveling in patient history [18, 23]. However, since 16s rDNA is unable to distinguish between the *F. tularensis* subspecies [10] the possibility of other subspecies as a causative agent cannot fully be ruled out.

The initially negative screening result with meningitis/encephalitis PCR panel combined with CSF Gram staining result (Gram-negative rod), colony morphology on agar plates, local increase of positive tularemia cases led us to consider *F. tularensis* as a cause for the infection. In addition, the time of the year and geographic location in an area of relatively high prevalence of *F. tularensis* also supported this possibility. As a matter of fact, during the time patient was at the UH, 10 antibody positive samples out of 32 studied (31%) were observed in the laboratory. It is interesting that all blood cultures remained negative in our patient case. It remains a possibility that longer culture (6 or 7 days) instead our routine 5.5-day incubation might have enabled the growth of *F tularensis*. However, this would not have affected the clinical decision making in our patient case, but it does underline the importance of PCR-based identification of slow growing bacteria, such as Brucella in clinical laboratories. As a matter of fact, our laboratory does offer clinicians the possibility to ask for extended culture if needed.

The delay in identifying the bacteria, was partly caused by the inability of MALDI-Tof Vitek MS to identify the bacterial culture in routine clinical laboratory. Retrospectively, the in-house Francisella PCR could have been utilized on CNS upon Gram staining finding and we have now added to our SOP Francisella PCR if initial CNS PCR remains negative and Gram-negative rod is found in the CNS Gram staining. Fortunately, the patient recovered completely, and traditional antibiotic susceptibility testing played an important role in discovering the appropriate treatment option; the initially unidentified bacteria was found to be sensitive to ciprofloxacin which is in line with most of the *F. tularensis* meningitis cases described in the literature [7, 9, 12, 16, 24]. Several groups have also studied the in vitro antibiotic sensitivity of *F. tularensis* [4, 5, 17]. These comprehensive studies have shown that fluoroquinolones, aminoglycosides, and tetracyclines are all effective for *F. tularensis* as in our case.

*F. tularensis* is difficult to isolate from clinical samples [18]. It could be argued, that *F tularensis* should be added also to the Vitek Maldi-tof library as it may cause severe infection -and also pose a risk of laboratory acquired infections to the staff if careful good laboratory practices are not followed. In our laboratory, all positive blood cultures bottles and tests on cultured bacterial plates are performed in laboratory hoods [19]. Interestingly, Bruker Maldi-tof does identify *F. tularensis*, though only in species level [20]. Recently, de Vries et al. [8] made Maldi-tof library validation to identify *F. tularensis*.

After patient recovery, the case was analyzed in a joint meeting of hospital infectious diseases medical staff and laboratory. *F tularensis* is not a common causative bacterium for meningitis and as expected incidence of meningitis caused by *F. tularensis* is low [7, 9, 12, 16, 24], the treatment was not initially directed towards *F. tularensis.* The resistance of our unidentified bacteria against antibiotics towards Gram-negative rods in our laboratory left relatively little choices for antimicrobial medication, namely ciprofloxacin and tobramycin and for this ciprofloxacin was successfully administered.

Overall, we noted here a possible pitfall in diagnosing *F. tularensis* as a causative agent for meningitis as the commercial version of Vitek Maldi-tof does not recognize this pathogen. A good knowledge of bacteriology, surveillance of local epidemiological situation and close collaboration between clinicians and the laboratory was needed for successful bacterial identification, appropriate antibiotic treatment and above all, complete patient recovery. Our case illustrated also a need to apply more specific nucleic acid based detection methods for rarer infectious agents such as *F tularensis*. We are currently planning a modification to our laboratory SOP to facilitate and increase the use of *F tularensis* PCR in case epidemiological and clinical situation suggests *F tularensis* might be the causative agent behind the symptoms of a patient.

## Data Availability

“All data and material are available for competent researchers from the corresponding author.”
